# Epidemiologic and clinicopathologic evaluation of patients with breast cancer referred to Ghaem Hospital from 2005 to 2014

**Published:** 2015

**Authors:** S Kadkhodayan, F Homaee Shandiz

**Affiliations:** * Department of Obstetrics and Gynecology, Women’s Health Research Center, Ghaem Hospital, Mashhad University of Medical Sciences, Mashhad, Iran,; ** Department of Radiotherapy Oncology, Research Center of Solid Tumor Treatment, Ghaem Hospital, Mashhad University of Medical Sciences, Mashhad, Iran

**Keywords:** cancer of breast, Triple negative, overall survival, disease free survival

## Abstract

**Introduction:** The most common cancer is Breast Cancer and the first principal purpose of cancer deaths in females of 44-40 ages. The currency of three negative breast cancer involves 10-17%. his sort cancer of the breast is described by a negative receptor of estrogen, progesterone, and HER2 that is much more competitive than the other kinds and the forecast is weaker. Therefore, this research was conducted with the aim of evaluating the outcomes of the medication in patients with a triple negative sort of breast cancer about the different patients with breast cancer.

**Method:** This historical group research was conducted by leading to the reports of all patients with breast cancer whose medication and follow-up was conducted in Hospital of Mashhad Ghaem through 2001 and 2010, their ER, PR, HER2 outcomes being reported in the files. Based on immunohistochemical records (ER, PR, HER2), patients were split into 2 collections: triple negative and another negative and the therapeutic results were analyzed among the 2 groups regarding two and five-year disease-free and throughout durability by Kaplan-Meier method. P<0.05 was regarded necessary.

**Results:** The medicinal studies of 331 cases with breast cancer were investigated in this research. The number of members in the Triple negative collection was 101 (30.5%) and in another negative group was 230 (69.5%). The base overall durability in the triple negative was 32.48 ± 24.56 months and in another negative was 29.67 ± 22.36 months and no notable variation was recognized in the studied collections (P=0.306). Furthermore, the low disease-free durability in the triple negative group was 30.57 ± 24.56 months and in another negative was 28.21 ± 21.72 months and no important variation was recognized in the studied collections (P=0.184).

**Conclusion:** under previously directed investigations, between all the samples of breast cancer, Triple negative had a weaker forecast and a lower durability with the cases in our research, the overall durability and disease-free longevity got were the same in 2 collections of Triple negative and another negative, and the basis of this relationship was apparently the residence of HER2 + subgroup in the non-Triple negative collection which pointed to the durability of cases in the non-Triple negative collection be similar to the Triple negative collection.

## Introduction

Breast cancer with a hidden purpose has represented the physicians’ care in all times. Notwithstanding hundreds of scientific ideas and methods, breast cancer is one of the most horrible human infections. Notwithstanding all the works were done for surgery, sadly, has no satisfying conclusion in some samples [**1**, **2**]. This current cancer is an enormous difficulty in the women’s health universal. Breast cancer is the most prevalent cancer in females and the 2nd other reason of death because of cancer in USA [**3**]. 

It was prophesied in 2009 that breast cancer covered 27% of all cancers and 15% of cancer-related death, covering 192,370 new cases and 40,170 cases of death [**1**]. Statistics and data indicated an increasing rate of breast cancer in the center of the 1940 decade [**3**, **4**].

The malignant increase of epithelial cells following the breast channels or lobules are the basis of this disease [**2**].

The core gauge biopsy and FNA procedures are the most popular breast cancer characteristic methods which can be applied to make all diagnostic and prognostic analyses with great outcomes [**2**]. Now, breast cancer is molecularly separated into 4 classes covering Luminal A, Luminal B, Her2 +, and Triple negative. However, between all sorts of breast cancer, the triple negative example includes 10-17%. This kind is recognized by negative receptor estrogen, progesterone, and HER2 that seems to be more competitive than another sort and has a weaker diagnosis [**4**,**5**].

Another study was retrospectively conducted by Abu al-Khair and associates in Saudi Arabia, in 2012, on 517 cases of cancer of breast, who were assigned to the Prince Abdul Aziz medical center from the first month of 2001 to the last month of 2008, the rate of Triple negative cancer in this area being related to the investigations in the West and having no meaningful distinction among the 2 collections in 3-year durability time. However, the aggressiveness and overall strength of the disease in the Triple negative collection was necessary for the samples of below than 40 ages than in those higher 40 years [**6**].

In the case of breast cancer also to general medical and surgical treatments (chemotherapy, radiotherapy, etc.), the operation depends on biological, and molecular tags of the tumor and the case (such as hormone treatment and purpose treatment) are further suggested [**7**,**8**]. The observed elements mentioned above show the value of the extra knowledge of this kind of cancer. Consequently, this research was conducted with the purpose of assessing the outcomes of therapy in cases with the triple negative type of breast cancer with different cases with breast cancer and their correlation with the overall durability and disease-free durability.

## Materials and Methods

This historical group research was done by leading to the reports of all cases of breast cancer whose therapy and follow-up was done in Hospital of Mashhad Ghaem during 2001 and 2010 and their ER, PR, and HER2 outcomes were listed in the files. Essential information was obtained and listed in the checklist and was investigated by a statistician. Essential information was obtained and listed in the checklist and was investigated by a statistician. Besides, the cases in the 2 collections were equaled regarding the disease’s step and therapy class.

The example size was estimated at 59 characters based on the research of Zaky et al [**9**] about the 70% and 90% durability rates in collections with and without Triple negative breast cancer with a confidence of 95% and 80% capacity. 

Composition principles involved all cases with breast cancer whose therapy and follow-up were conducted in Mashhad Ghaem Hospital during 2001 and 2010 and their ER, PR; HER2 outcomes were listed in the files. Exclusion tests involved non-carcinoma pathology, the cases who had no formal leading for the therapy and follow-up (at shortest 6 months), and the metastatic samples.

The analyzed variable involved years, breast cancer model (Triple negative and another negative), tumor pathology, infection stage, disease grade, the basic and middle and percentage of overall durability and disease-free longevity in Triple-negative and another negative collection, simultaneous effect of age and illness stage and disease grade in the overall durability and disease-free durability.

The charts and mathematical tables were applied to explain the information. Chi-square test was applied to assess the association between the qualitative changeable (years< and > forty years, the tumor diagnostics, infection step, disease category, recurrence) with Triple negative and another negative (Luminal A, Luminal B, HER2 +).

ANOVA analysis was applied to assess the association between years and Triple negative and another negative (Luminal A, Luminal B, HER2 +) and Log-rank was univariate applied to determine the relationship between Triple negative and another negative (Luminal A, Luminal B, HER2 +), and the overall durability and disease-free longevity and the rate and mean of overall durability and disease-free survival in any Triple negative and non-Triple negative collections (Luminal A, Luminal B, HER2 +) was received by the Kaplan-Meier system.

Cox regression was also applied to estimate the contemporary influences of changeable. In all analyses, SPSS application version 16 was applied and P<0.05 was determined important.

## Results

In this research, medical reports of 331 cases of breast cancer which led to the Oncology Center of Ghaem Hospital for therapy and follow-up through 2001 and 2010 were assessed. Based on the immunohistochemistry outcomes possible in the file, members were split into 2 Triple negative (TN) and another negative collections based on the immunohistochemistry about the ER and PR, the cases of > 1% were reported as positive and the ones of < 1%, negative, and, about HER2, only the cases of three positive were reported as positive. Some members in the Triple negative collection was 101 (30.5%) and in another negative collection was 230 (69.5%). Furthermore, the members of another negative collection were split into 3 subtypes of luminal A, luminal B and HER2 + the number of cases in any collection was 142 (42.9%), 36 (10.9%) and 52 (15.7%), individually.

The standard age of members in the TN collection was 43.50 ages, and in the another negative collection it was 48.08 ages, the conclusion of the one-way analyses confirming that the order of age was not related to the prepared collections and the cases with Triple negative cancer were younger (P=0.001). The number of cases aged <40 ages was reached in 43 samples (42.6%) in the TN collection and of 64 cases (27.8%) in the another negative collection, the outcome of the Chi-square analysis is revealing a meaningful distinction among the examined collections the pattern of years which was not related to the processed collections and the number of cases aged <40 years in the TN collection was necessary related to the another negative collection (P=0.008).

The number of cases with carcinoma ductal attack in the TN collection was of 83 samples, medullary carcinoma 8, lobular carcinoma 2, and another sample 5. The kind of cancer was obscure in 3 samples of Triple negative collection and 5 in another negative collection, the outcome of the Chi-square analysis revealing a meaningful distinction among the examined collections and the number of different kinds of cancer which was not related to the investigated collections and the subgroup of medullary breast carcinoma was more seen in the Triple negative collection (P=0.042).

In the TN selection, some members in step 1 were of 10 samples, in step 2 it was of 59, in step 3 it was of 32, the outcome of the Chi-square analysis confirming that the number of the stage was related to Triple negative and another negative collection (P=0.945). 

In the TN selection, some of the members in class was of 0 cases, and in another negative collection, it was of 22 samples. The data compared to the class was not possible for 63 members in the Triple negative collection and 131 in another negative collection, the outcome of the Chi-square analysis confirming that the number of class was not related to the Triple negative and another negative collection; no cases with Triple negative breast cancer in class A were seen (P=0.006).

The standard overall durability in members with TN breast cancer was of 32.48 ± 24.1 months, and in the another negative collection, it was of 29.67 ± 22.36 months. The overall 5-year durability time in members with TN breast cancer was 71% and in the non-Triple negative collection was 76.5%. The outcome of Log-rank analysis revealed no important variation in disease-free durability among the examined collections (P=0.306) (**Table 1**).

**Table 1:** Comparison of the overall survival among Triple negative and another negative groups

Variables		Groups		P-value
		Non TN	TN	
Mean overall survival		22.36 ±29.67	32.48 ± 24.1	0.306
Median of survival		22.5	27	
Overall survival (%)	1 year	99	96.7	
	2 years	91.2	92.6	
	5 years	76.5	71	

**Fig. 1:**
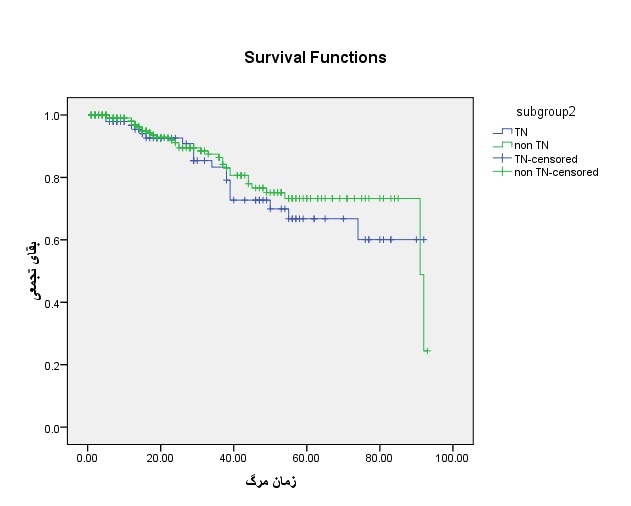
Comparison of the overall survival in Triple negative and non-Triple negative groups

The mean disease-free survival in patients with TN breast cancer was of 30.57 ± 24.56 months and in the non-Triple negative group, it was of 28.21 ± 21.72 months. The five-year disease-free survival in patients with TN breast cancer was 66.7% and in the non-Triple negative group, it was 73.3%. The result of Log-Rank test showed no significant differences in the disease-free survival among the studied groups (P=0.184) (**Table 2**).

**Table 2:** Comparison of the disease-free survival in the Triple negative and non-Triple negative groups

Variables		Groups		P-value
		Non TN	TN	
Mean disease-free survival		28.21 ± 21.72	30.57 ± 24.56	0.184
Median of survival		21	20	
Disease-free survival (%)	1 year	95.9	92.4	
	2 years	89.9	79.8	
	5 years	73.3	66.7	

**Fig. 2:**
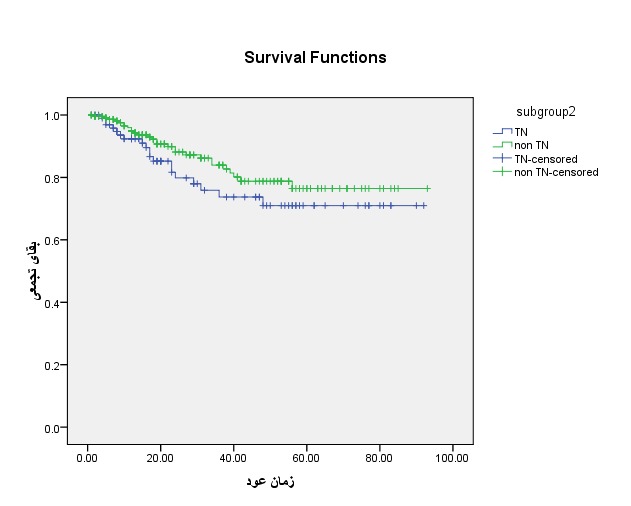
Disease-free cumulative survival in the Triple negative and non-Triple negative groups

Cox regression was used to evaluate simultaneous effects of grade, stage, age, and the studied groups on disease-free survival and the result is shown in **Table 3**.

**Table 3:** Results of Cox regression to assess the simultaneous effect of variables on the disease-free survival

Variables	Confidence interval 95%	Risk ratio	Model coefficient	P-value
Stage 1	-	1ª	-	0.620
Stage 2	(0.113, 8.672)	0.988	-0.012	0.992
Stage 3	(0.020, 5.811)	0.343	-1.070	0.459
Age	(0.394, 5.618)	1.488	0.397	0.558
Grade 1	-	1ª	-	0.996
Grade 2	(0.000,-)	0.000	-12.218	0.979
Grade 3	(0.291,3.828)	1.055	0.054	0.935
a=reference group				

Cox regression results showed that among the factors of age, stage, grade, and the studied groups, none is independently associated with disease-free survival.

Also, the Cox regression was used to evaluate simultaneous effects of grade, stage and age on the overall survival rate and the result is shown in **Table 4**.

**Table 4:** Results of Cox regression to assess the simultaneous effect of variables on the overall survival

Variables	Confidence interval 95%	Risk ratio	Model coefficient	P-value
Stage 1	-	1ª	-	0.804
Stage 2	(0.167, 11.151)	1.363	0.310	0.773
Stage 3	(0.067, 9.547)	0.797	-0.227	0.858
Age	(0.266, 3.761)	1.000	0.000	1.000
Grade 1	-	1ª	-	0.900
Grade 2	(0.000,-)	0.000	-12.398	0.979
Grade 3	(0.216, 2.594)	0.748	-0.290	0.647
a=reference group				

Cox regression results showed that among the factors of age, stage, and grade, there were no elements independently associated with the overall survival.

## Discussion 

Cancer is the main difficulty about the health of the world. Between all kinds of cancer with females, breast cancer is the most prevalent. Given that half of the world’s crowd are females, so several crowds in the world are in danger of growing this kind of cancer. Hence, more investigations in this region are required. This kind of cancer has different subtypes, between these, being the Triple negative sample which is recognized by the negative receptor of estrogen and progesterone and -HER2 defined by immunohistochemically methods, having a feeble diagnosis between cases [**10**,**11**].

As presented in earlier investigations, also to tumor subtypes, the cases’ age at examination, degree and class of illness, obesity, menopausal state, race, and lymph node involvement are more significant factors in the determination of diagnosis and durability of the cases [**12**, **13**].

This study investigated to cover the cases who had played the therapy suggested by the doctor and had at shortest six months of follow-up, the cases not being different at the end of the course of chemotherapy and radiotherapy, the outcome of the Chi-square analysis in the estimate of some these samples in the examined collection drawing the right selection of the members. Also, there was no variation among cases in this opinion, and the outcome of these samples on the diagnosis and durability of cases can be dismissed.

Regarding choosing the kind of therapy, just the number of hormone treatment was not related to the examined collection, which is because of the negative hormone receptor in the Triple negative collection, leading into account that hormone treatment could not be applied in the therapy of this collection.

In this research, the currency of Triple negative breast cancer was 30.5% with a base age of 43.50 years, which was more popular in females related to a survey conducted by Dent and associates in Canada. In the research of Dent and colleagues, the currency of Triple negative breast cancer was 11.2%, and the frequency of recurrence and death within 5 years from the analysis was extra than that of the non-Triple negative collection [**14**].

So, in the investigations led by Lin and colleagues in Taiwan [**15**], Zaky et al. in Atlanta [**16**], Davis and colleagues in West Virginia [**17**], the number of Triple negative breast cancer was weaker than in our research, but in the research of Sen and colleagues [**18**] in Calcutta, the number of Triple negative breast cancer was recorded as 27.78% which was statistically related to our research in Iran.

In a research by Davis and colleagues in West Virginia published in 2007, the pathological tumor subgroup of most members with Triple negative breast cancer was an invasive ductal carcinoma, which is related to our research, suggesting that the pathological tumor subset of 84.7% of members with Triple negative breast cancer was invasive ductal carcinoma [**17**].

In research by Sen and colleagues in the Department of Surgery, School of Medicine, reported in Calcutta in 2013, with cases with Triple negative breast cancer, 75% were in Step 3 and 80% in Class 3 of the illness but related to our research, this number was changed. In our research, 58.4% of the cases with Triple negative breast cancer were in Step 2 and 52.2% were in class 2 of their illness [**18**].

In this research, 2 and 5-year overall durability and 2 and 5-year disease-free durability were evaluated in the Triple negative and another negative collections by leading to the cases’ follow-up according to the satisfied of their medical reports, covering dates of per visit and controlling the state of cases (recurrence, death, good position) that were assessed and reported on per visit by the doctor, and were analyzed between cases by applying the Kaplan-Meier analysis. The outcomes were related to research by Abu al-Khair and colleagues in Saudi Arabia in which 26 members with at shortest three years follow-up in the Triple negative collection and 33 members with equal age and illness step in the non-Triple negative collection were chosen and the outcome of a 3-year durability was related in both collections [**6**]. n this current study, greatest (57.4%) of the members with Triple negative breast cancer were aged > forty years, suggesting that the number of the both collections was not determined as the equal and the cases with Triple negative breast cancer were younger than those with non-Triple negative breast cancer, but according to the outcomes of Cox regression analysis, there was no meaningful correlation between age and overall durability and disease-free survival in the examined collections.

Also, in research conducted with Boyle in France, the greatest frequency of Triple negative breast cancer recurrence was through 3 years of analysis and the valuation of death was incredibly developed through 5 years of analysis and the African-American community was a danger factor in this cancer which in this class was 3 times more prevalent than another model of breast cancer. It can be assumed that maybe the cause of the variation in managing the overall durability in the cases of our research and the research of Boyle was the result of a race that needed more education in later [**19**].

## Conclusion

Based on past led investigations, between all the samples of breast cancer, the Triple negative has a weaker forecast and a lower durability with the members in our research, the overall durability and disease-free durability got was the equal in both collections of Triple negative and another negative, indicating that the basis of this relationship was apparently the attendance of HER2 + subgroup in the non-Triple negative collection, which has pointed to the durability of cases in another negative collection, which was related to the Triple negative collection.

If needed, medications useful on HER2 + receptor could be applied more in the therapy of members with another negative breast cancer. It is also suggested that in later investigations, menopausal state and obesity and its impact on the durability of the cases were viewed and the outcomes were associated with results of previous surveys since in our research, it was not the principal purpose and it was not decided. Furthermore, if extra investigations were played on the Triple negative breast cancer in Asia and including the evaluation of the ethnic state, we could have a better correlation of the number of this kind of cancer in the Asian and Western cultures.
